# Leukaemia cutis as a late, isolated extramedullary relapse: a case report of a patient with acute myeloid leukaemia 6 years after allogeneic bone marrow transplantation and remission

**DOI:** 10.1093/skinhd/vzaf070

**Published:** 2026-02-10

**Authors:** Fatemeh Montazer, Sina Karaji, Nima Dastgir

**Affiliations:** Department of Pathology, Firoozabadi Hospital, School of Medicine, Iran University of Medical Sciences (IUMS), Tehran, Iran; Department of Pathology, School of Medicine, Iran University of Medical Sciences (IUMS), Tehran, Iran; Department of Infectious disease, Hashemi Nejad Hospital, Tehran, Iran

## Abstract

Leukaemia cutis (LC) is characterized by the infiltration of leukaemic cells into skin tissue, presenting as various skin lesions. It can signal relapse or coexist with extramedullary or systemic relapses. Extramedullary relapse, particularly in sanctuary sites like the skin, remains a clinical challenge, demanding investigation into mechanisms and monitoring strategies. We present a unique case of isolated extramedullary relapse manifesting as LC, 6 years after successful allogeneic bone marrow transplantation for acute myeloid leukaemia. A 24-year-old man exhibited a cutaneous nodule on his scalp, prompting comprehensive evaluation. Despite achieving complete remission post-transplantation, a core needle biopsy confirmed LC. Extensive assessments, including blood tests and bone marrow biopsy, returned normal results. As the disease was confined to skin, the patient received local radiotherapy. This case instead highlights the overall challenge of detecting and treating late, isolated extramedullary relapse.

## Teaser

Leukaemia cutis (LC) occurs when leukaemic cells infiltrate the skin, signalling relapse or coexisting with systemic disease. Extramedullary relapse, particularly in sanctuary sites like the skin, presents a clinical challenge. We report a rare case of isolated LC relapse 6 years post-allogeneic bone marrow transplant for acute myeloid leukaemia. A 24-year-old male developed a scalp nodule, which biopsy confirmed as LC, despite normal blood and marrow results. He received local radiotherapy. This case highlights the need for prolonged vigilance and research to improve post-transplant relapse detection and management.

What is known about this topic?

- Leukaemia cutis is a rare manifestation of extramedullary relapse and often signals systemic recurrence in patients with acute myeloid leukaemia (AML).
- Isolated extramedullary relapse after haematopoietic stem cell transplantation is uncommon and presents diagnostic and management challenges.

What does this study add?

- This case highlights a late, isolated skin relapse of AML occurring 6 years post-transplant, without bone marrow involvement.
- It emphasizes the importance of long-term surveillance and supports the role of site-directed therapy, such as radiotherapy, in managing solitary cutaneous relapse.

Leukaemia cutis (LC), a phenomenon characterized by the infiltration of leukaemic cells into skin tissue, typically presents as discernible skin lesions or nodules. Skin lesions may appear as papules, nodules, plaques or patches anywhere on the body. Notably, the sites of LC encompass diverse anatomical locations, including the breast, testis, skin and subcutaneous tissues. It is important to recognize that skin involvement might serve as an initial indicator of relapse or coexist with other extramedullary or systemic relapses.^1,2^

While post-bone marrow transplantation relapse remains a significant challenge in the management of patients with acute myeloid leukaemia (AML), it is noteworthy that extramedullary relapse appears to occur in approximately one-third of cases. Furthermore, instances of isolated extramedullary relapse, devoid of bone marrow involvement, appear to be even less frequent. Specifically, a subset of isolated extramedullary relapses occurs within the skin tissue.^3^

Late relapse, characterized by recurrence after a minimum of 5 years of remission, is an infrequent occurrence, accounting for only 1–3% of patients with AML.^4^ Isolated extramedullary relapse is predominantly documented in patients with AML, with rarer occurrences noted in other myeloproliferative disorders such as chronic myeloid leukaemia, and a tendency towards a higher prevalence in male patients.^5^ Although extramedullary relapse following transplantation in patients with AML in remission and without systemic involvement has conventionally been regarded as the initial sign of the commencement of a renewed systemic relapse, isolated extramedullary involvement has also been observed.^6^

We present an exceptional and distinctive case that showcases a rare instance of leukaemia relapse. Specifically, our case involves a late and isolated extramedullary relapse manifesting in the skin, occurring 6 years following allogeneic bone marrow transplantation.

## Case report

**Figure 1:**
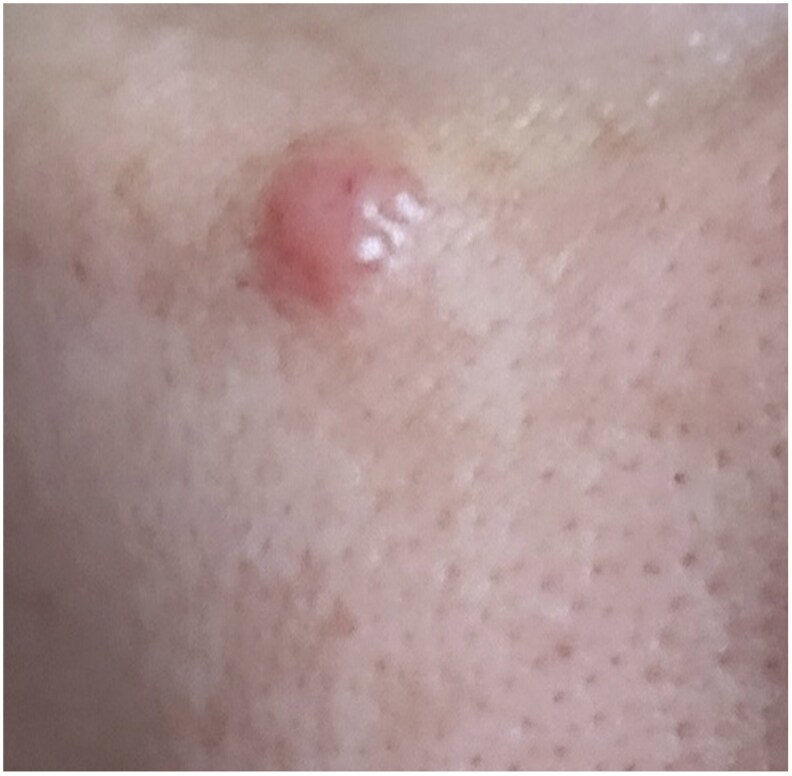
A solitary skin-coloured firm and painless nodule with a smooth surface.

A 24-year-old man presented with a cutaneous nodule located on the left frontal scalp. A scalp nodule developed over 1 month, without systemic symptoms (Figure 1).

Six years previously, the patient was diagnosed with AML and underwent a successful allogeneic bone marrow transplantation at an outside hospital. He achieved complete remission and remained asymptomatic. Regular follow-up appointments, including two bone marrow biopsies with normal results, had been conducted since his recovery.

**Figure 2:**
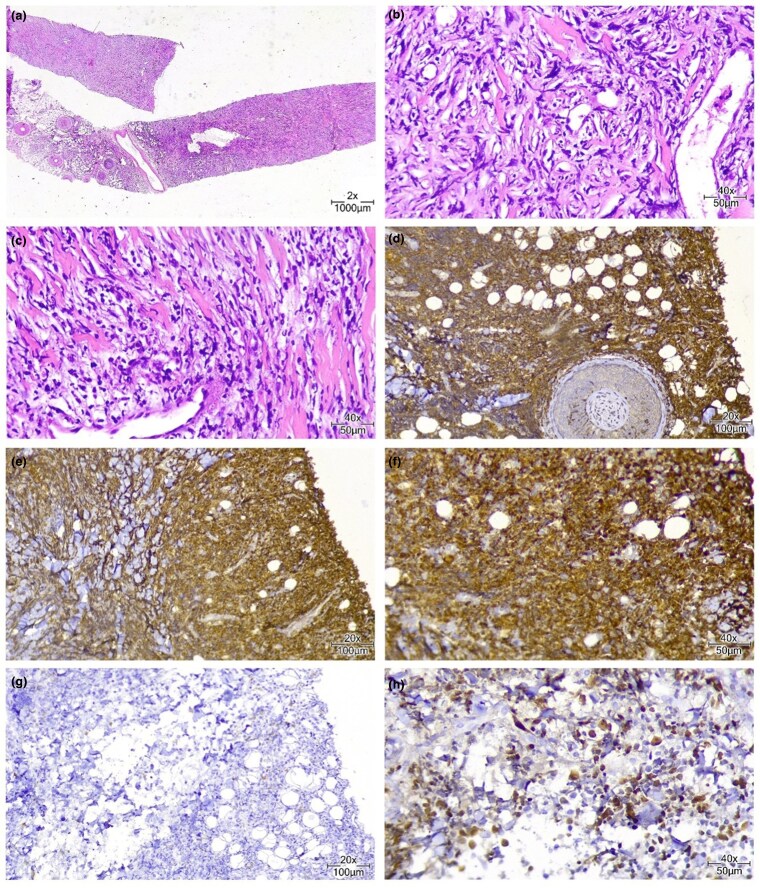
Histopathological and immunohistochemical analysis of scalp skin mass. (a) Core needle biopsy of scalp skin mass: haematoxylin and eosin (H&E) staining, low-power view, revealing a cellular tumoral lesion. (b, c) H&E staining, high-power views, highlighting pleomorphic tumoral cells infiltrating skeletal muscle bundles. (d) Immunohistochemistry (IHC) staining for CD117, demonstrating positive expression in most tumoral cells. (e) IHC staining for CD68, indicating positive expression. (f) IHC staining for myeloperoxidase, displaying positive expression. (g) IHC staining for CD20, showing negative expression. (h) IHC staining for Ki-67, revealing positive expression of the proliferation marker.

A core needle biopsy was performed on the cutaneous nodule, confirming the diagnosis of LC. Microscopic examination revealed a dense diffuse/interstitial infiltrate involving the dermis and subcutis, sparing the papillary dermis (grenz zone) and epidermis (Figure 2). Perivascular and periadnexal involvement were also observed. Stromal fibrosis, crush artefact and an Indian file pattern of growth were noted. The neoplastic infiltrate consisted of medium-to-large mononuclear cells with scant cytoplasm and slightly basophilic nuclei with visible nucleoli. Some of these neoplastic cells had elongated (spindle) and kidney-shaped nuclei, and there were also atypical cells with clear cytoplasm and intracytoplasmic granules. Scattered mitoses were evident.

Immunohistochemical analysis gave a positive Ki-67 result with a high proliferation index, indicating increased cellular activity (Table 1). CD45 staining confirmed the presence of leukaemic cells with positive staining in some. CD34 was moderately positive in most cells. Strong myeloperoxidase expression indicated a myeloid nature; CD68 was also positive in tumour cells. These findings provided critical insights into the patient’s condition, aiding in the diagnostic process and guiding potential treatment strategies.

**Table 1:** Immunohistochemistry results

Parameter	Result
MPO	Strongly positive in all suspected cells
CD68	Weak-to-moderate positive staining in most suspected cells
CD45	Positive in some suspected cells
CD56	Weak-to-moderate staining in most suspected cells
TdT	Positive in some suspected cells (20%)
Ki-67	Intermediate-to-high proliferation index in suspected cells
CD3	Negative in suspected cells
CD20	Negative
CD4	Negative in suspected cells
CD34	Moderately positive staining in most suspected cells
CD43	Strongly positive in the majority of suspected cells
CD117	Moderately positive staining in most suspected cells

MPO, myeloperoxidase.

Following the diagnosis, due to concerns about systemic relapse, the patient underwent a comprehensive workup, including blood tests and bone marrow biopsy, all of which returned normal results. The patient was subsequently referred to a haematology–oncology referral centre for consideration of local radiotherapy.

Lymph node testing was clear, indicating the absence of any abnormal or cancerous involvement in the lymphatic system. The patient was further referred to a specialized AML centre, and no new symptoms have been reported since the diagnosis up until submitting this report.

## Discussion

This case is notable for the rare occurrence of skin involvement following transplantation, especially without systemic relapse or blood-related issues. However, a new challenge has emerged as the patient experienced LC. This demands a thorough exploration of possible mechanisms and careful consideration of prolonged post-transplant monitoring and care.

Extramedullary relapse, although uncommon, has important clinical implications post-bone marrow transplantation. Its presentation is often deceptive, especially without systemic relapse. For example, renal relapse may cause abdominal pain;^7^ tumour-related neural compression can lead to limb pain and paraesthesia;^8^ hearing loss and otalgia may mimic acute otitis media; and cardiac involvement can result in pericardial effusion.^9,10^

The exact occurrence of extramedullary relapse after haematopoietic stem cell transplantation (HSCT) remains uncertain. However, on study investigated 436 patients with AML who underwent HSCT. Of 128 patients who relapsed after HSCT, 25 experienced relapse in extramedullary sites.^11^ These relapses, occurring exclusively in the skin of five patients, manifested distinctly, transpiring approximately 5 months after the instances of patients with bone marrow relapse. Remarkably, the study highlights a significant 6-month survival advantage following relapse in patients with isolated extramedullary relapse, as opposed to those with combined extramedullary and bone marrow relapse or bone marrow relapse alone. Investigators propose that subsequent to HSCT, extramedullary sites might conceivably act as secure havens for quiescent leukaemic cells. This sheltering effect could protect these cells from therapeutic interventions and immune reactions, thereby fostering a conducive milieu for their incremental expansion.^11^

LC serves as a distinctive dermatological hallmark exclusive to extramedullary AML. This unique presentation is characterized by the infiltration of leukaemic cells into the layers of the epidermis, dermis or subcutaneous tissue, accentuating its clinically significant nature.^12^ In the context of LC, leukaemic cells, typically found in the bloodstream, can infiltrate the skin through interactions with specific molecules on blood vessel walls. These interactions allow the cells to attach, migrate through vessel walls and accumulate in the skin. After bone marrow transplantation, while healthy cells restore blood and immune systems, leukaemic cells in extramedullary tissues may persist. Leukaemic cells’ migratory and adhesive features, coupled with tissue-specific signalling, can prolong their presence post-transplant. Treatments used during transplantation target rapidly dividing cells, sparing dormant leukaemic cells and enabling survival in extramedullary sites. Immune clearance might be less efficient outside the bone marrow.^13^

Graft-versus-tumour effect refers to the beneficial response where the transplanted immune cells recognize and attack cancer cells remaining in the recipient’s body. This effect is desired in the context of stem cell transplantation as it can contribute to the elimination of residual cancer cells and prevent relapse. In another study, researchers reported a patient who experienced isolated cutaneous extramedullary relapse. Similar to our patient, she did not show systemic engagement. Following the patient’s stem cell transplantation, she received immunosuppressive treatment, including tacrolimus, to prevent graft-versus-host disease (GvHD). The strategy of discontinuing immunosuppression in pursuit of enhancing the immune response against the tumour aligns with the objective of achieving more effective and targeted therapeutic outcomes. The authors highlighted that although achieving complete remission and chimerism in the bone marrow is possible, attaining chimerism at the tissue level and subsequently inducing a graft-versus-leukaemia effect might be limited.^14^ This highlights the need for a more detailed investigation into extramedullary relapses, especially in relation to previous instances of GvHD and the potential impact of immunosuppressive treatments administered.

This late, isolated skin relapse 6 years post-HSCT highlights the need for extended follow-up and site-specific therapy. The phenomenon of extramedullary relapse, especially in sanctuary sites like the skin, necessitates further research to enhance our understanding and therapeutic strategies for managing such cases effectively.

## Data Availability

All data are available in the article.
